# Characterisation of Muffins with Upcycled Sunflower Flour

**DOI:** 10.3390/foods10020426

**Published:** 2021-02-15

**Authors:** Simona Grasso, Tatiana Pintado, Jara Pérez-Jiménez, Claudia Ruiz-Capillas, Ana Maria Herrero

**Affiliations:** 1 Institute of Food, Nutrition and Health, School of Agriculture, Policy and Development, University of Reading, Reading RG6 6AH, UK; 2 Institute of Food Science, Technology and Nutrition (ICTAN-CSIC), 28040 Madrid, Spain; tatianap@ictan.csic.es (T.P.); jara.perez@ictan.csic.es (J.P.-J.); claudia@ictan.csic.es (C.R.-C.); ana.herrero@ictan.csic.es (A.M.H.)

**Keywords:** muffins, by-product, valorisation, sunflower flour, amino acid profile, antioxidant activity, mineral content, fibre content, FRAP, PCL assay

## Abstract

There is an increased interest and need to make our economy more circular and our diets healthier and more sustainable. One way to achieve this is to develop upcycled foods that contain food industry by-products in their formulation. In this context, the aim of this study was to develop muffins containing upcycled sunflower flour (a by-product from the sunflower oil industry) and assess the effects of sunflower flour addition on the fibre, protein, amino acid, mineral content, and antioxidant activity measured by a Ferric Reducing Antioxidant Power (FRAP) assay and Photo chemiluminescence (PCL) assay. Results show that the sunflower flour inclusion significantly improved all the parameters analysed as part of this study. A more balanced muffin amino acid profile was achieved, thanks to the increased levels of lysine, threonine, and methionine, the limiting essential amino acids of wheat flour. We can conclude that upcycled ingredients, such as sunflower flour, could be used for the nutritional improvement of baked goods, such as muffins. Their addition can result in several nutritional advantages that could be communicated on packaging through the use of the appropriate EU nutrition claims, such as those on protein, fibre, and mineral content.

## 1. Introduction

In order to make our economies more circular and our diets more sustainable, there is an increased need to valorise food industry by-products into ingredients that can re-enter the food chain as part of new foods.

Sunflower cake is a by-product of the sunflower oil industry which has been traditionally used as animal feed [1]. Rich in protein, fibre, essential amino acids and minerals [1], it has been reported to have a high antioxidant potential [2]. Recently, sunflower cake has been upcycled into a functional flour by a US start-up through the patented use of novel technologies, such as extrusion and steam explosion [3], which have opened up this under-valorised ingredient to a whole new range of food applications. Researchers have so far used it on both baked goods [4,5] and meat product applications [6], reporting promising results.

Circular economy principles should push us to valorise food industry by-products as ingredients for human diets, rather than just as animal feed, as explained in the food recovery hierarchies developed in the EU and in US [7,8]. This is especially relevant if we consider that food industry by-products contain several nutrients of interest, such as protein, fibre, minerals and vitamins. We know that the demand for proteins will continue to increase in the future [9]; therefore, valorising food industry by-products rich in proteins could be a positive step towards sustainable protein production. Similarly, in 2015, the Scientific Advisory Committee on Nutrition brought the recommended daily intake of fibre to 30 g, while the average intake in adults is around 18 g of fibre daily [10]; therefore, fibre-rich by-products could play a key role in meeting this nutritional need when suitably incorporated into new foods.

Popular baked goods, such as muffins, cakes, or biscuits, are usually high in sugar and fat but low in fibre, antioxidants, and minerals [11], so they could represent ideal foods to be reformulated to be healthier through the use of upcycled ingredients. Efforts to include ingredients such as pecan nut meal, spent coffee, and several fruit and vegetable pomaces [4,12,13,14] in muffins have recently been reported, showing an increased research interest in this area.

The aim of this short communication was to partially replace wheat flour with sunflower flour (at 15% or 30%) in muffins and evaluate the effects of this replacement on their fibre, mineral, and amino acid content, as well as antioxidant activity. No attempts have been made so far to investigate these parameters in the development of muffins with upcycled sunflower flour, while an investigation on the proximate composition (moisture, ash, protein, and fat), physical analyses, and sensory quality of muffins with sunflower flour can be found in a study by Grasso, Liu, and Methven [4].

## 2. Materials and Methods

### 2.1. Muffin Manufacture

Muffins were manufactured according to the recipe and procedure shown by Ateş and Elmacı [15] and the ingredients reported by Grasso, Liu, and Methven [4]. Muffins with sunflower flour were prepared by replacing wheat flour with sunflower flour at either 15% or 30% [4]. The following ingredients made up 100 g of control dough: 28.2 g sugar, 24.4 g wheat flour, 20.7 g whole egg, 15.8 g sunflower oil, 8.6 g water, 1.2 g skimmed milk powder, 0.9 g baking powder, and 0.2 g salt. Experimental muffins were prepared by replacing wheat flour with sunflower flour at either 15% (3.7 g/100 g) or 30% (7.3 g/100 g) [4]. Briefly, egg and sugar were mixed for 1 min with a Kenwood Hand Mixer (HM520, Reading, UK) at low speed. Then, oil, milk powder in water, flour only (for control muffins) or flour and sunflower flour (for 15% and 30%), baking powder, and salt were added. The ingredients were mixed for 3 min at high speed. Batter portions of 40 g ± 0.5 g were baked in paper muffin cases placed onto muffin trays in batches of 12 units in a pre-heated, ventilated oven (Kwick_Co, Salva, Gipuzkoa, Spain) for 20 min at 190 °C. After 1 h of cooling time, the muffins were kept in sealed plastic bags to prevent moisture loss.

### 2.2. Dietary Fibre Content

Dietary fibre content was evaluated according to Grasso, Pintado, Pérez-Jiménez, Ruiz-Capillas, and Herrero [6]. Duplicate measurements were carried out for each sample, and results were expressed as g/100 g of sample.

### 2.3. Protein Content

Protein content was measured in duplicate with a Nitrogen Determinator LECO FP-2000 (Leco Corporation, St Joseph, MI, USA). The factor used to convert nitrogen content to protein was 6.25, and the results were expressed as g/100 g of the sample.

### 2.4. Mineral Content

For mineral content determination, freeze-dried samples (Lyophilizer Telstar-Cryodos Equipment, Tarrasa, Spain) were prepared by acid digestion with nitric acid in a microwave digestion system (ETHOS 1, Milestone, Srl, Sorisole, Italy). The minerals were quantified on a ContrAA 700 High-Resolution Continuum Source spectrophotometer (Analytik Jena AG, Jena, Germany) equipped with a Xenon short-arc lamp (GLE, Berlin, Germany). Three determinations were carried out per sample to measure Calcium (Ca), Magnesium (Mg), Sodium (Na), Potassium (K), Phosphorus (P), Iron (Fe), Zinc (Zn), Copper (Cu), and Manganese (Mn). The determinations were made in duplicate, and the results were expressed as mg/100 g of the sample. More information on the mineral content analysis can be found in a study by Sánchez-Faure et al. [16].

### 2.5. Amino Acid Content

Amino acid content was determined and measured using ninhydrin derivative reagent and separated by means of cation-exchange chromatography, using a Biochron 20 automatic amino-acid analyser (Amersham Pharmacia Biotech. Biocom, Uppsala, Sweden) where we injected the extract of samples that was dried and hydrolysed in vacuum-sealed glass tubes at 110 °C for 22 h in the presence of 6 N HCl containing 0.1% phenol and nor leucine (Sigma Aldrich, Inc.) as the internal standard. After hydrolysis, samples were again vacuum-dried, dissolved in application buffer, and injected onto a Biochrom 20 amino-acid analyser (Pharmacia, Barcelona, Spain). A mixture of amino acids was used as the standard (Sigma Aldrich, Inc., Madrid, Spain). The determinations were made in duplicate, and the results expressed as mg/g of the sample.

### 2.6. Antioxidant Activity

For the determination of antioxidant capacity, an aqueous-organic extraction was carried out in duplicate following the methodology of Jiménez et al. [17].

#### 2.6.1. Ferric-Reducing Antioxidant Power (FRAP) Assay

FRAP reagent, freshly prepared and warmed to 37 °C, was mixed (150 μL) with distilled water (15 μL) and the test sample, Trolox, or appropriate blank solvent (5 μL). Readings at 595 nm in a Synergy MX (BioTek, Madrid, Spain) spectrophotometer after 30 min were selected to calculate the FRAP values. Results were expressed as µg eq Trolox/mg after interpolating in the calibration curve.

#### 2.6.2. Photo Chemiluminescence (PCL) Assay

This assay was used to determine antioxidant capacity using an automated photo chemiluminescent system (Photochem, Analytik Jena Model AG; Analytic Jena USA, The Woodlands, TX, USA), which measures the capacity to quench free radicals. This method is based on controlled photochemical generation of radicals, part of which is quenched by the antioxidant, and the remaining radicals are quantified by a sensitive chemiluminescence-detection reaction. Results were expressed as µg eq Trolox/mg sample (liposoluble fraction) and µg eq ascorbic acid/mg (hydro soluble fraction).

### 2.7. Statistical Analysis

The baking experiment was repeated twice on two different days. One-way analysis of variance (ANOVA) was carried out to evaluate differences between formulations using the SPSS program (v.22, IBM SPSS Inc., Chicago, IL, USA). To compare mean values between formulations, least squares differences and Tukey’s HSD tests were used to identify significant differences (*p* < 0.05) between formulations.

## 3. Results and Discussion

The fibre and protein content of the sunflower flour and the three muffins are shown in Table 1. Soluble dietary fibre was below the limit of detection, and this was expected, due to the mainly insoluble nature of the fibre reported in sunflower by-products [2]. The insoluble dietary fibre and protein content increased with increasing sunflower flour inclusion. Both muffins with sunflower flour provide at least 3% fibre; therefore, they would represent “a source of fibre”, according to the current EU regulations [18]. This is a positive result, as food industry by-products could be used as ingredients to enhance the nutritional content of baked goods, such as muffins, as recently shown with spent coffee grounds [13] and grape pomace [19].

The mineral content of the sunflower flour and the muffins is shown in (Table 2). Sunflower oil cake, on a dry basis, contains 0.48 g/100 g calcium, 0.84 g/100 g phosphorus, 0.44 g/100 g magnesium, and 3.49 g/100 g potassium [20]. As a result of the sunflower flour inclusion, all minerals subject to analysis significantly increased (except for sodium), and both 15% and 30% muffins can be considered “a source of” or “high in” several minerals, according to the current EU regulations [21]. The 15% and 30% muffins could be considered a source of magnesium (>56.2 mg/100 g) and manganese (>0.4 mg/100 g), as well as high in phosphorous (>210 mg/100 g). The 30% muffins could be considered a source of potassium (>300 mg/100 g), iron (>2.2 mg/100 g), and zinc (>1.6 mg/100 g). Finally, the 15% muffins can be considered a source of copper (>0.2 mg/100 g), and the 30% muffins can be considered high in copper (>0.4 mg/100 g). Mehta et al. [22] also reported a significant mineral content increase with the addition of tomato pomace in bread and muffins, so food industry by-products could be used as ingredients to increase the micronutrient value of appropriately reformulated baked goods.

Table 3 shows the results of the amino acid analysis performed on the sunflower flour and the muffins. For four non-essential amino acids (aspartic acid, glycine, alanine, arginine) and three essential amino acids (valine, methionine, leucine), the addition of sunflower flour resulted in a significant amino acid increase, compared to the control (30–60% increase between control and 30% muffins). Additionally, for these amino acids, the 30% muffins showed significantly higher content than the 15% muffins. For four amino acids (essential threonine, isoleucine, phenylalanine, and non-essential tyrosine), there was a significant amino acid increase only between the control and 30% muffins (increase in the range 27–39%), but the amino acid content was similar between the control and 15% muffins. For the non-essential amino acids glutamic acid and proline, there was no significant difference in terms of content across the three muffins, while cysteine was the only amino acid where a non-significant decrease was recorded in sunflower muffins compared to the control. For the essential amino acids histidine and lysine, the addition of sunflower flour resulted in similar levels in the 15% and 30% muffins, and in lower levels in the control muffins (26–33% increase between control and 30% muffins). Finally, the content of the non-essential amino acid serine was highest in the 30% muffins and lowest in the control muffins, while the 15% muffins had an intermediate serine content and were not significantly different from the control or 30% muffins. The addition of distillers’ grain flour was reported to improve the amino acid content of muffins, especially the levels of threonine, serine, glutamic acid, alanine, methionine, leucine, and histidine [23]. As reported by Siddiqi et al. [24], the amino acid composition of wheat is quite unbalanced, lacking the essential amino acids lysine, threonine, and methionine. Since sunflower flour addition increased the content of these amino acids lacking in wheat, the incorporation of sunflower flour could help to achieve a more balanced amino acid profile in muffins.

Table 4 shows the Food and Agriculture Organization (FAO) adult amino acid requirements [25], the amino acid content of sunflower flour, and the amino acid score of sunflower flour. The amino acid score determines the effectiveness with which absorbed dietary nitrogen can meet the indispensable amino acid requirement at the safe level of protein intake [25]. This is achieved by a comparison of the content of the amino acid in the protein with its content in the requirement pattern [25]. It can be seen that the first limiting amino acid in sunflower flour is lysine, while all the other sunflower amino acids have a score of at least one, and up to almost two.

Table 5 shows the results of the antioxidant capacity tests carried out on the muffins. The addition of sunflower flour resulted in a dose-dependent significant increase in the antioxidant activity of the muffins, with the 30% muffins showing significantly higher antioxidant capacity than the 15% muffins, with the 15% muffins showing higher values than the control muffins. Previous results on biscuits with sunflower flower also showed an increased antioxidant capacity through the 2,2-Diphenyl-1-picrylhydrazyl (DPPH) assay and the cupric reducing antioxidant capacity (CUPRAC) assay [5], which was related to the higher total phenolic content of the sunflower flour compared to wheat flour. It has been shown that sunflower meal is a good source of phenolic compounds with high antioxidant capacity (such as chlorogenic, caffeic, p-hydroxybenzoic, p-coumaric, cinamic, m-hydroxybenzoic, vanillic, syringic, transcinnamic, isoferulic, and sinapic acids [26]), while wheat flour has a very low polyphenol content [27]. An increase in the natural antioxidant content of baked goods could help in terms of shelf life by lowering the oxidation of fats and would help to keep the food as a “clean label” [28].

## 4. Conclusions

The use of upcycled ingredients in baked goods, such as sunflower flour in muffins, could result in several nutritional advantages as here shown, such as improved fibre content, mineral content, amino acid profile, and antioxidant activity. The development of baked goods with a balanced amino acid profile through the use of upcycled ingredients is of particular interest and should be explored in further research. Upcycled ingredients could be promoted on the packaging if they are used at sufficient levels to make nutrition claims, such as those on fibre, protein, or mineral content. The sensory quality of muffins with sunflower flour was investigated through a Quantitative Descriptive Analysis by Grasso, Liu, and Methven [4]. Results showed that the 15% muffins were the most similar to the control, and that further reformulation was needed to improve the sunflower samples. Future efforts should also concentrate on developing recipes that are healthier overall (for example, by using less sugar in the batter). A holistic and multi-disciplinary approach should be used in the development of such novel baked goods, considering several aspects at once, such as the nutritional profile, as well as sensory and technological aspects, to create new foods that deliver in taste and that will be well-received by consumers..

## Figures and Tables

**Table 1:** Dietary fibre and protein content (g/100 g of sample) of sunflower flour and muffins.

	Sunflower Flour	Control	15%	30%
Soluble dietary fibre	1.84 ± 0.15	<LOD	<LOD	<LOD
Insoluble dietary fibre	24.55 ± 1.67	2.27 ± 0.17 ^c^	3.60 ± 0.10 ^b^	4.58 ± 0.17 ^a^
Protein	30.99 ± 0.16	7.08 ± 0.11 ^c^	8.33 ± 0.12 ^b^	9.52 ± 0.07 ^a^

LOD: limit of detection. Data are expressed as means ± standard deviation (*n* = 4). Different letters indicate significant differences (*p* < 0.05) for the same analysis.

**Table 2:** Mineral content (mg/100 g sample) of sunflower flour and muffins.

Mineral	Sunflower Flour	Control	15%	30%
Calcium	54.00 ± 1.16	65.45 ± 1.63 ^c^	78.50 ± 2.62 ^b^	97.21 ± 3.11 ^a^
Magnesium	64.17 ± 1.91	16.44 ± 1.24 ^c^	68.34 ± 7.67 ^b^	106.4 ± 4.93 ^a^
Sodium	2.04 ± 0.04	400.0 ± 24.9 ^a^	397.5 ± 14.8 ^a^	407.7 ± 18.8 ^a^
Potassium	213.0 ± 14.5	137.6 ± 2.85 ^c^	203.1 ± 7.64 ^b^	303.1 ± 6.51 ^a^
Phosphorus	70.39 ± 3.99	195.4 ± 10.6 ^c^	223.7 ± 10.2 ^b^	254.7 ± 9.87 ^a^
Iron	1.48 ± 0.02	1.01 ± 0.12 ^c^	1.56 ± 0.19 ^b^	2.49 ± 0.28 ^a^
Zinc	1.41 ± 0.00	0.74 ± 0.03 ^c^	1.25 ± 0.02 ^b^	1.80 ± 0.02 ^a^
Copper	0.31 ± 0.00	0.17 ± 0.01 ^b^	0.23 ± 0.01 ^b^	0.43 ± 0.01 ^a^
Manganese	0.38 ± 0.00	0.24 ± 0.01 ^c^	0.49 ± 0.02 ^b^	0.78 ± 0.03 ^a^

Data are expressed as means ± standard deviation (*n* = 4). Different letters indicate significant differences (*p* < 0.05) for the same mineral among muffins.

**Table 3:** Amino acid content (mg/g sample) of sunflower flour and muffins, and percentage amino acid content change between sunflower flour muffins and control.

	Amino Acid	Sunflower Flour	Control	15%	% Change 15%-Control	30%	% Change 30%-Control
Non-essential amino acids	Aspartic acid	24.66 ± 0.28	6.30 ± 0.37 ^c^	7.58 ± 0.13 ^b^	+20	8.82 ± 0.17 ^a^	+40
	Serine	11.67 ± 0.62	5.57 ± 0.29 ^b^	5.79 ± 0.05 ^ab^	+4	6.30 ± 0.07 ^a^	+13
	Glutamic acid	49.63 ± 2.21	18.43 ± 1.11 ^a^	19.51 ± 0.32 ^a^	+6	20.74 ± 0.44 ^a^	+13
	Proline	17.55 ± 0.81	7.88 ± 0.30 ^a^	8.47 ± 0.01 ^a^	+7	8.53 ± 0.22 ^a^	+8
	Glycine	14.42 ± 0.31	2.76 ± 0.16 ^c^	3.78 ± 0.05 ^b^	+37	4.49 ± 0.04 ^a^	+63
	Alanine	11.55 ± 0.26	3.69 ± 0.19 ^c^	4.51 ± 0.03 ^b^	+22	5.03 ± 0.02 ^a^	+36
	Cysteine	2.28 ± 0.11	0.86 ± 0.11 ^a^	0.66 ± 0.02 ^a^	-23	0.72 ± 0.01 ^a^	-16
	Tyrosine	7.39 ± 0.24	1.36 ± 0.13 ^b^	1.36 ± 0.04 ^b^	0	1.76 ± 0.11 ^a^	+29
	Arginine	16.19 ± 0.45	2.15 ± 0.07 ^c^	2.64 ± 0.06 ^b^	+23	3.45 ± 0.23 ^a^	+60
Essential amino acids	Valine	11.71 ± 0.33	3.65 ± 0.12 ^c^	4.06 ± 0.08 ^b^	+11	4.74 ± 0.05 ^a^	+30
	Methionine	4.67 ± 0.14	0.73 ± 0.09 ^c^	1.05 ± 0.11 ^b^	+44	1.33 ± 0.10 ^a^	+82
	Isoleucine	9.80 ± 0.29	2.35 ± 0.15 ^b^	2.69 ± 0.09 ^b^	+14	3.27 ± 0.06 ^a^	+39
	Leucine	16.86 ± 0.47	4.51 ± 0.03 ^c^	4.89 ± 0.14 ^b^	+8	5.89 ± 0.16 ^a^	+31
	Threonine	10.01 ± 0.28	3.05 ± 0.16 ^b^	3.31 ± 0.06 ^b^	+9	3.88 ± 0.06 ^a^	+27
	Phenylalanine	14.23 ± 0.38	3.05 ± 0.30 ^b^	3.30 ± 0.06 ^b^	+8	4.12 ± 0.18 ^a^	+35
	Histidine	7.24 ± 0.33	2.05 ± 0.12 ^b^	2.49 ± 0.02 ^a^	+21	2.72 ± 0.07 ^a^	+33
	Lysine	10.66 ± 0.41	4.33 ± 0.36 ^b^	5.13 ± 0.07 ^a^	+18	5.47 ± 0.12 ^a^	+26

Data are expressed as means ± standard deviation (*n* = 4). Different letters indicate significant differences (*p* < 0.05) for the same amino acid among muffins.

**Table 4:** Adult FAO amino acid requirements, amino acid content, and amino acid score of sunflower flour.

Amino Acid	Adult Requirement (FAO) mg/g Protein	Sunflower Flour mg/g Protein	AA Score Sunflower Flour
Methionine + cysteine	22	22.43	1.02
Isoleucine	30	31.62	1.05
Leucine	59	54.40	0.92
Threonine	23	32.30	1.40
Phenylalanine + tyrosine	38	69.76	1.84
Histidine	15	23.36	1.56
Lysine	45	34.40	0.76
Valine	39	37.79	0.97

**Table 5:** Antioxidant capacity of muffins evaluated by FRAP (Ferric Reducing Antioxidant Power) and PCL (photo chemiluminescence).

	Control	15%	30%
FRAP (µg eq Trolox/mg sample)	1.52 ± 0.16 ^c^	2.99 ± 0.17 ^b^	4.36 ± 0.36 ^a^
PCL—liposoluble (µg eq Trolox/mg sample)	* nd	0.44 ± 0.04 ^b^	1.20 ± 0.13 ^a^
PCL—hydrosoluble (µg eq ascorbic acid/mg sample)	0.04 ± 0.02 ^c^	6.04 ± 0.25 ^b^	18.79 ± 1.07 ^a^

* nd: not detected. Data are expressed as means ± standard deviation (*n* = 4). Different letters indicate significant differences (*p* < 0.05) for the same analysis.

## Data Availability

Not applicable.
